# The role and function of noise and neural heterogeneity in the integrated population response of the vestibulo-ocular reflex

**DOI:** 10.1186/1471-2202-12-S1-P127

**Published:** 2011-07-18

**Authors:** James McGuinness, Bruce P Graham

**Affiliations:** 1 Institute of Computing Science and Mathematics, School of Natural Sciences, University of Stirling, Stirling, Scotland, FK94LA, UK

## 

We investigate a functional role and cause for the neural variability in the integrated population response of the linear component of the Horizontal Vestibulo-Ocular Reflex (VOR), specifically looking at the role and function of noise and neural heterogeneity in this system.

The linear component of the VOR is produced through an elementary 3 neuron reflex arc, and is characterised by short latency and high fidelity at frequencies up to 20Hz. However, single cell responses of Medial Vestibular Nucleus (MVN) neurons become non-linear and distorted for inputs greater than 12Hz, and a homogeneous population becomes highly synchronised when a common input is applied, and so neither can adequately encode the required information. It has therefore been theorised that an asynchronous population is required to achieve the necessary wide frequency response of the VOR [1].

**Figure 1:**
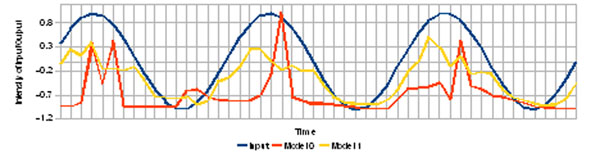
Population responses for homogeneous & noiseless MVN population (Model 0), and heterogeneous & noisy population (Model1) for an input of 50pA at 12Hz, produced by current injection.

We use previously published, compartmental models of (A & B subtype) MVN neurons [2] incorporated into the NEURON Simulation Environment to investigate the affect of noise and heterogeneity in producing and maintaining asynchronicity of the population, and their affects on the fidelity of response produced by the population (see Figure 1). We provide results from 2 stages of simulations: a 1st stage utilizing a current injection as an abstraction of both the common input to the population, and the variance in spontaneous (or 'pacemaker') activity in the population; and a 2nd stage in which we model synaptic input as the common input to members of the population, and model heterogeneity through variance in the density of mechanisms responsible for the spontaneous activity of the neurons across the population. These simulations are performed over a range of input frequencies and amplitudes.

It is shown that a heterogeneous population, where each member shows a different rate of spontaneous activity, produces a response with greater fidelity than a homogeneous population. In addition, different distributions of variability in spontaneous activity, different input amplitudes, and different intensities of noise, show different response fidelities, suggestive of a form of optimization in the MVN populations.
